# Assessment of the relationship between obesity and the level of anhedonia and self-assessment of mental state in patients with schizophrenia

**DOI:** 10.1192/j.eurpsy.2026.12160

**Published:** 2026-07-31

**Authors:** A. Borowska, K. Kowalczyk, M. Wadoń, Z. Mielczarek, M. Reclik, K. Krysta, B. Trędzbor

**Affiliations:** 1 Department and Clinic of Psychiatric Rehabilitation, Faculty of Medical Science in Katowice, Medical University of Silesia, Katowice; 2 Clinical Psychiatric Hospital in Rybnik, Rybnik; 3 Students’ Scientific Association, Department and Clinic of Rehabilitation Psychiatry, Faculty of Medical Sciences in Katowice, Medical University of Silesia , Katowice, Poland

## Abstract

**Introduction:**

The lifespan of patients with schizophrenia is significantly shorter compared to the general population. One of the reasons is the high prevalence of metabolic syndrome in this group. Treatment with neuroleptics reduces levels of dopamine, the main neurotransmitter in the reward system. Researchers have explored links between obesity, anhedonia, and the severity of clinical symptoms of schizophrenia.

**Objectives:**

To assess the relationship between obesity and the level of anhedonia and self-assessment of mental state in patients with schizophrenia.

**Methods:**

Anthropomorphic assessment (height, weight, waist circumference, blood pressure) was performed in two groups of patients with schizophrenia:those with normal weight: BMI < 25 (n=22) and those with obesity: BMI > 30 (n=21).

Patients completed scales assessing the level of anhedonia (SHAPS) and the Severity of Schizophrenia Symptoms Scale (FBS).

Statistical analysis was performed.

**Results:**

The distribution of the presented data was determined to be different from normal in the Shapiro-Wilk test performed, which had a power (alpha) of 5%, with a p-value of <0.05. Therefore, the purpose of the analysis of variables was to use the non-parametric Mann-Whitney U test, resulting in values: p=0.048.

The relationship between normal BMI and BMI in the obesity range and the number of points on the SHAPS scale is statistically significant (Graph 1).

The distribution of the presented data was determined to be different from normal in the Shapiro-Wilk test performed, which had a power (alpha) of 5%, with a p-value of <0.05. Therefore, the purpose of the analysis of variables was to use the non-parametric Mann-Whitney U test, resulting in values: p=0.771.The relationship between normal BMI and BMI in the obesity range and the number of points on the FBS scale is not statistically significant (Graph 2). Conclusion drawn from the analysis:

**Image 1:**
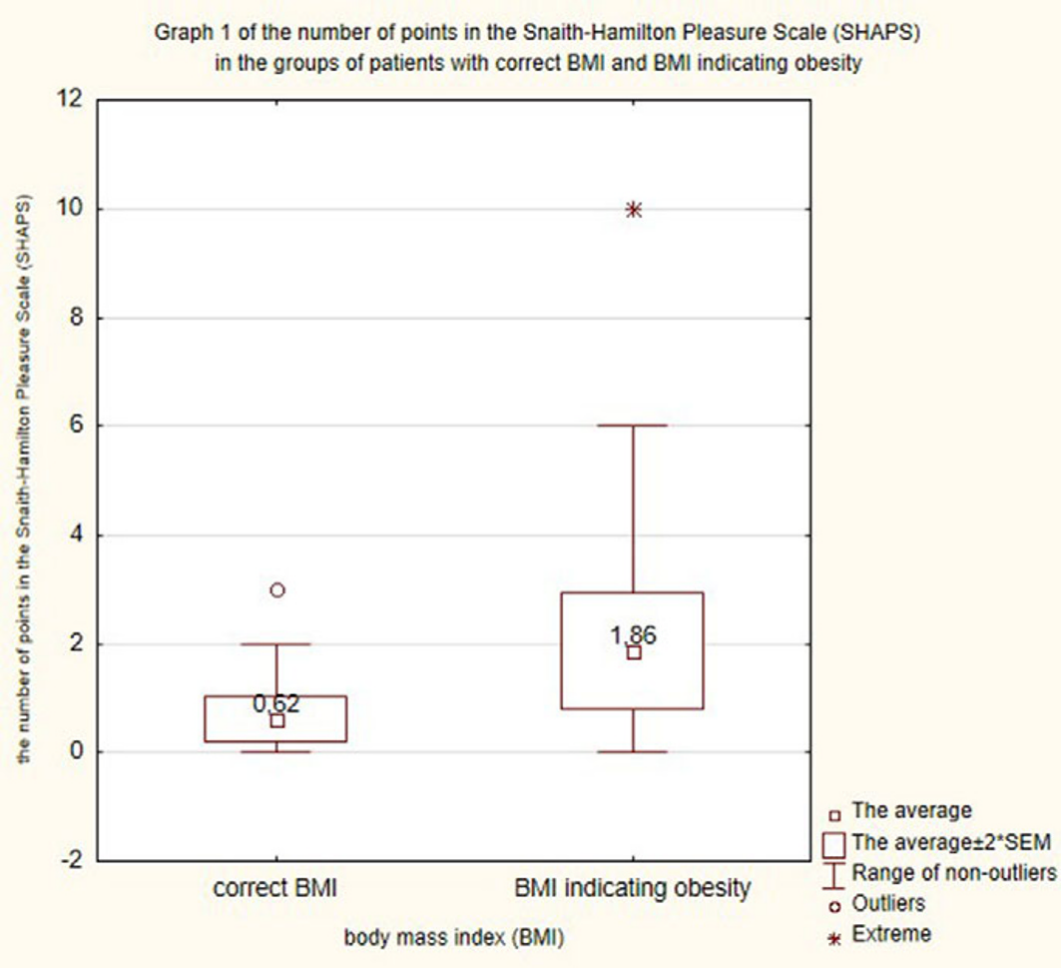
Image 1: Long description.

**Image 2:**
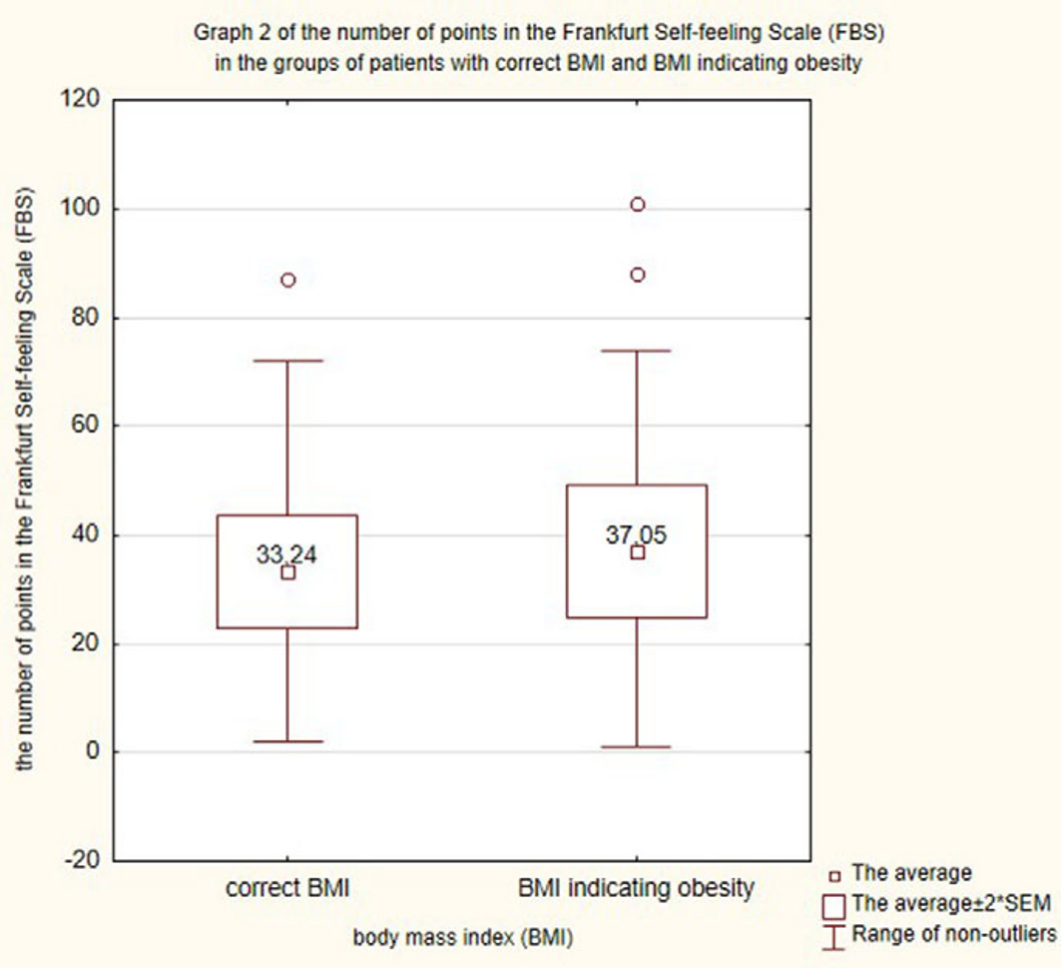
Image 2: Long description.

**Conclusions:**

Patients with obesity exhibit a greater number of symptoms of anhedonia compared to patients with normal body weight.

Patients with obesity do not exhibit a greater number of symptoms on the FBS scale compared to patients with normal body weight.

**Disclosure of Interest:**

None Declared

## Long descriptions

### Image 1: Long description

The Y-axis is labeled the number of points in the Snaith-Hamilton Pleasure Scale S H A P S and ranges from -2 to 12 in increments of 2. The X-axis is labeled body mass index B M I and contains two categories.

1. Correct B M I. The average is marked at 0.62. The box representing the average plus or minus 2 times S E M spans from approximately 0.2 to 1.0. The range of non-outliers extends from 0 to 2. One outlier is represented by a circle at 3.

2. B M I indicating obesity. The average is marked at 1.86. The box representing the average plus or minus 2 times S E M spans from approximately 0.8 to 3.0. The range of non-outliers extends from 0 to 6. One extreme value is represented by an asterisk at 10.

A legend in the bottom-right defines the symbols. A small square represents the average. A larger rectangle represents the average plus or minus 2 times S E M. An I-shaped bar represents the range of non-outliers. A circle represents outliers. An asterisk represents extreme values.

Navigate back to Image 1:.

### Image 2: Long description

The Y-axis represents the number of points in the Frankfurt Self-feeling Scale F B S ranging from negative 20 to 120. The X-axis categorizes patients by body mass index B M I.

* The first group on the left is correct B M I. It shows an average of 33.24 represented by a small square. The box representing the average plus or minus 2 times S E M spans from approximately 23 to 44. The range of non-outliers extends from near 0 to 72. One outlier is plotted as a circle near 87.

* The second group on the right is B M I indicating obesity. It shows a higher average of 37.05. The box representing the average plus or minus 2 times S E M spans from approximately 25 to 49. The range of non-outliers extends from near 0 to 74. Two outliers are plotted as circles at approximately 88 and 101.

A legend in the bottom right corner defines the symbols: a small square for the average, a large box for the average plus or minus 2 times S E M, and error bars for the range of non-outliers.

Navigate back to Image 2:.

